# Larger body size leads to greater female beluga whale ovarian reproductive activity at the southern periphery of their range

**DOI:** 10.1002/ece3.8367

**Published:** 2021-11-23

**Authors:** Steven H. Ferguson, David J. Yurkowski, Justine M. Hudson, Tera Edkins, Cornelia Willing, Cortney A. Watt

**Affiliations:** ^1^ Fisheries and Oceans Canada Freshwater Institute Winnipeg Manitoba Canada; ^2^ Biological Sciences University of Manitoba Winnipeg Manitoba Canada

**Keywords:** age, body length, *Delphinapterus leucas*, fitness, geographic range, ovarian corpora

## Abstract

Identification of phenotypic characteristics in reproductively successful individuals provides important insights into the evolutionary processes that cause range shifts due to environmental change. Female beluga whales (*Delphinapterus leucas*) from the Baffin Bay region (BB) of the Canadian Arctic in the core area of the species’ geographic range have larger body size than their conspecifics at the southern range periphery in Hudson Bay (HB). We investigated the mechanism for this north and south divergence as it relates to ovarian reproductive activity (ORA = total corpora) that combines morphometric data with ovarian corpora counted from female reproductive tracts. Our study aim was to assess the relative influence of age and body size of female beluga whale on ORA in the two populations. Female beluga whale ORA increased more quickly with age (63% partial variation explained) in BB than in HB (41%). In contrast, body length in HB female beluga whales accounted for considerably more of the total variation (12% vs. 1%) in ORA compared to BB whales. We speculate that female HB beluga whale ORA was more strongly linked with body length due to higher population density resulting in food competition that favors the energetic advantages of larger body size during seasonal food limitations. Understanding the evolutionary mechanism of how ORA varies across a species’ range will assist conservation efforts in anticipating and mitigating future challenges associated with a warming planet.

## Graphical abstract

Beluga whales in the eastern Canadian Arctic group into a core region in the north and a peripheral region in the south composed of smaller whales. We test for the relative contribution of size while controlling for age and find that the peripheral beluga whale body length is more strongly linked to ovarian reproductive activity (total corpora) than the core whales. We consider the ecological and evolutionary explanations for this difference and how it may relate to shifts in geographic range with warming.

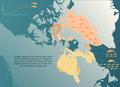

## 1 INTRODUCTION

Evolution occurs through natural selection whereby individuals with greater fitness contribute disproportionately more genetic information to future generations. In addition to this individual variation, populations will vary due to adaptations to different environmental selection pressures (Coulon et al., 2008; Orsini et al., 2008; Pauls et al., 2013). Population‐level differences in average fitness could then vary geographically along an environmental gradient, such as altitude or latitude, based on location within the species’ range (Kirkpatrick & Barton, 1997; Peterson et al., 2011). For example, sink populations at the periphery of a species’ range are constantly in phenotypic flux due to the demographic challenges of living in an environment where species‐specific traits are less well adapted compared to populations near the core of the species’ range (Gaston, 2009; Sheth & Angert, 2016). It is critical to understand the extent of species‐level plasticity that allows individuals to track extreme environmental selection pressures at the edge of their geographic range in our rapidly changing world to inform conservation (Hardie & Hutchings, 2010; Valladares et al., 2014).

Populations at the core of the species’ range, where individuals are presumably most suitably adapted to their environment, likely differ from populations at the range periphery where greater phenotypic variation occurs. Reproductive activity is costly and offers a potentially relevant metric to assess the suitability of females to their environment. The ovaries of many mammals provide an index of ovarian reproductive activity (ORA; Ellis et al., 2018; Marsh et al., 1984) and therefore researchers have used lab examinations of female reproductive tracts from sustainably hunted individuals to identify the number of ovarian corpora (Lehmann, 1993; Nazarova & Evsikov, 2012; Ringsby et al., 2009). Whales are distinct in that their corpora albicantias (CA) physically remain for the duration of the whale's life, providing a possible way to track an individual's historical record of reproductive events and number of lifetime ovulations (Ellis et al., 2018; Perrin et al., 1984). As a result, we can examine the ovarian reproductive history of individual whales since each CA represents one ovulation, although not necessarily a pregnancy (Berta et al., 2015). During ovulation, an oocyte is released from the Graafian follicle with the rupture site forming the corpus luteum (CL), a temporal bright yellow, hormonal gland helping to promote and to maintain implantation of the embryo. Subsequently, this body regresses to a CA, which is generally considered to persist within the ovarian tissue throughout the life of a female whale even after diminishing in size and color (Fujino, 1963; Laws, 1961; Mackintosh, 1942).

Relating phenotypic characteristics to lifetime reproductive activity can provide important insight into evolutionary processes and allow comparisons between populations that may indicate adaptation (Peterson et al., 2019). We thus need to assess the contribution of variation in phenotypic traits, such as body size, to reproductive variation (Gaillard et al., 2000), in order to understand key variables for survival and reproductive success.

Large mammalian females are generally considered to be capital breeders (Stearns, 1992) and, therefore, should illustrate a strong relationship between individual body size and reproductive activity. Despite relationships between reproductive metrics and body size being investigated across several mammalian species (pinnipeds (Le Boeuf & Reiter, 1988), ungulates (Clutton‐Brock et al., 1988), and rodents (Ribble, 1992), this relationship has not been demonstrated in whales, likely due to the logistical difficulties of measuring adult body size and reproduction over an individual's lifetime. Odontocete (toothed) whales generally live in cooperative societies where selection on female dominance likely operates through variation in body size (Croft et al., 2017; Ward et al., 2009). No studies have investigated how age and body mass interplay to shape variation in female ORA in odontocete whales.

**FIGURE 1:**
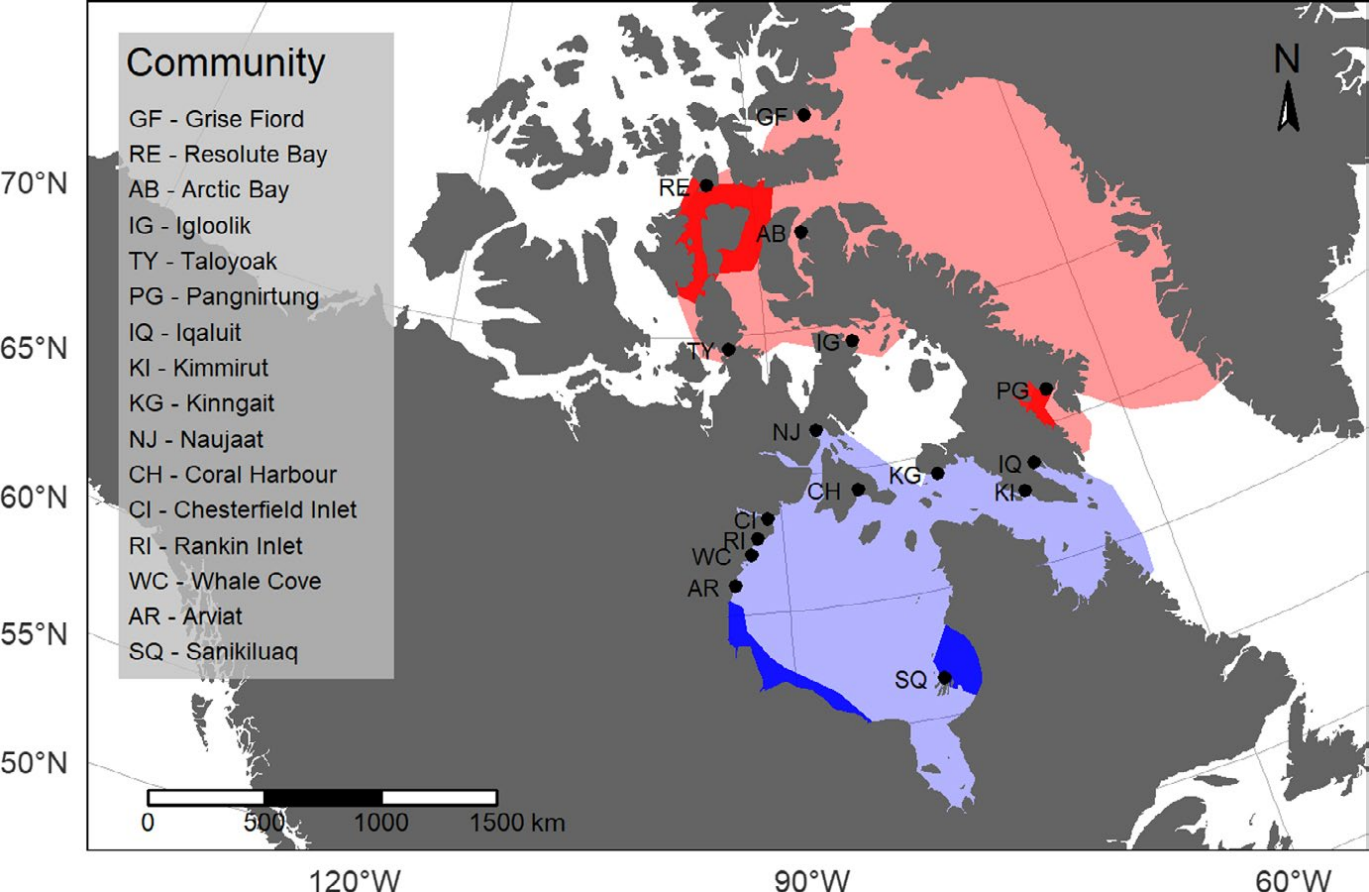
Study area delineating the two regions representing core (BB–red) and peripheral (HB–blue) beluga whale populations and the 16 Nunavut, Canada communities where sampling took place. Darker colors represent summer range that is used to define the populations

There are 21 beluga whale (*Delphinapterus leucas*) populations across the Arctic providing a latitudinal continuum of populations within their range (Hobbs et al., 2020). A collection of tissue samples provided by Inuit hunters during subsistence hunts from across the eastern Canadian Arctic have been archived by Fisheries and Oceans Canada and include female beluga whale reproductive tracts with ovaries. To date, this collection has revealed spatial differences in morphology, phylogenetic history, demography, and reproduction between individuals wintering in the Hudson Bay (HB) region, compared to those wintering in Baffin Bay (BB) (Ferguson et al., 2020; Postma, 2017) (Figure 1). For this study, we chose to compare the HB whales, representing adaptations to life at the southern periphery of the beluga whale geographic range (59° latitude), to BB whales (73° latitude) representing adaptations to life within the core of the species’ range. Knowing that HB whales are smaller than BB whales (Stewart, 1994), our objective was to determine whether female body size differences relating to ORA occurred between peripheral HB and core BB regions while controlling for age. Specifically, we determined how variation in ORA, measured as total ovarian corpora counts, relates to body size of female beluga whales from both populations.

## 2 METHODS

The dataset included 172 female reproductive tracts with at least one corpus: 41 from BB and 131 from HB. To create a complete dataset required for robust statistical testing (Moritz & Bartz‐Beielstein, 2017), missing length and age data were replaced with the median value of all whales in each population. The five BB whales with missing age were assigned 20 years‐of‐age and the 6 HB whales, 26 years‐of‐age. Similarly, the 6 BB whales with missing length were assigned 354 cm and the 17 HB whales with missing length, 327 cm. We conducted postmortem gross examinations of female reproductive tracts, collected from 17 northern communities within the Eastern Canadian Arctic from 1989 to 2014 (Figure 1). Aging was based on examination of dentine and cementum growth layer groups in teeth (Waugh et al., 2018). Whale standard length was measured in the field according to a standard protocol, measured from the middle of the fluke to the tip of the rostrum (American Society of Mammalogists, 1961). We combined reproductive morphology data for two northern populations (Cumberland Sound and high Arctic) into a BB region based on a similar growth‐age‐reproduction relationship (Ferguson et al., 2020). For consistency in terminology, we refer to BB and HB as populations while recognizing that each region likely comprises a number of sub‐populations (Skovrind et al., 2021).

Sample processing is described in more detail in Ferguson et al. (2020); briefly, ovaries were excised, weighed, measured, and preserved in 10% neutral‐buffered formalin. For each ovary, we recorded the number of CLs and CAs (Best, 1968). In cetaceans CLs and CAs form distinct and persistent features, accumulating within the ovary (Perrin et al., 1976) as a record of a female's potential reproductive history (Slijper, 1962; Collet and Harrison, 1981; but see Dabin et al., 2008). Corpora assessments were performed by one reader to minimize bias in the subjective determination of accessory corpora (Harrison, 1977). As a measure of ORA, we counted all existing CLs and CAs within the beluga whales’ ovaries. For whales with only one ovary sampled (23 of 97 whales from BB and 113 of 210 whales from HB), we doubled the corpora count since beluga whales do not appear to exhibit a side‐dominance in ovarian function (Robeck et al., 2010; Shelden et al., 2019).

### 2.1 Statistical analysis

A Generalized Linear Mixed Model fit by maximum likelihood (Laplace approximation) with a Poisson distribution (Coxe et al., 2009) was used to assess differences in ORA between the two regions. Poisson regression models are best used for modeling events where the outcomes are counts or, more specifically, discrete data with non‐negative integer values. Generalized Linear Models are models in which response variables follow a distribution other than the normal distribution. Knowing that the relationships between ORA and age or body length are non‐linear (Lemaître et al., 2020), we transformed the non‐linear relationship to linear form using a link function creating a log‐linear model, whereby the coefficients are calculated using maximum quasi‐likelihood. Region (categorical), age (continuous), and length (continuous) were included as fixed effects and year as a random effect. Model selection was guided by Variance Inflation Factors and Akaike's information criterion for small sample size (AICc) using information theory (Burnham & Anderson, 2002). We calculated log‐likelihood, AICc values, ∆AICc, and AICc weights (wi – relative likelihood of the model) using MuMIn (version 1.43.17; Barton, 2020; Zuur et al., 2009). First (Step 1), we tested the full model to determine whether the effect of length and age on ORA differed by region. Then (Step 2), we addressed region‐specific relationships by removing region as a fixed effect and running separate models for each region. Our study employed a limited set of a priori models (i.e., n = 6), and therefore we report all top models (∆AICc < 3.0) while accepting that models with AIC scores near the top‐ranked model might not be as competitive based on consideration of model deviance (Arnold, 2010; Burnham & Anderson, 2002). All statistical analyses and graphics were performed using R statistical software (v. 3.6.3).

The effect of body size on ORA was assessed for each region separately while controlling for whale age. We used partial correlations, which measured the “unique” contribution of an independent variable (age and body length) to *r* ^2^ of the model. Here, we followed the “hierarchical analysis procedure” where the order of variable entry affected analysis and interpretation of partials (Cohen & Cohen, 1975). As a result, length was the first predictor variable entered into the model, due to length being the primary variable of interest to answer our hypotheses, followed by age. The partial correlation analysis assumes linearity in the relationships among ORA, age, and length, which we tested with residual plots (Zuur et al., 2010). To display possible nonlinearities, we used LOESS (locally estimated scatterplot smoothing) in the figures as a non‐parametric regression method that combines multiple regression models in a k‐nearest‐neighbor‐based meta‐model (Owolabi et al., 2016). To test for differences in slope and interecept between the ORA relationship with age and length, we used linear analysis of covariance. To express effect size we used Cohen's *d* = (Mean1 – Mean2)/Standard Deviation (Cohen, 1969).

## 3 RESULTS

**FIGURE 2:**
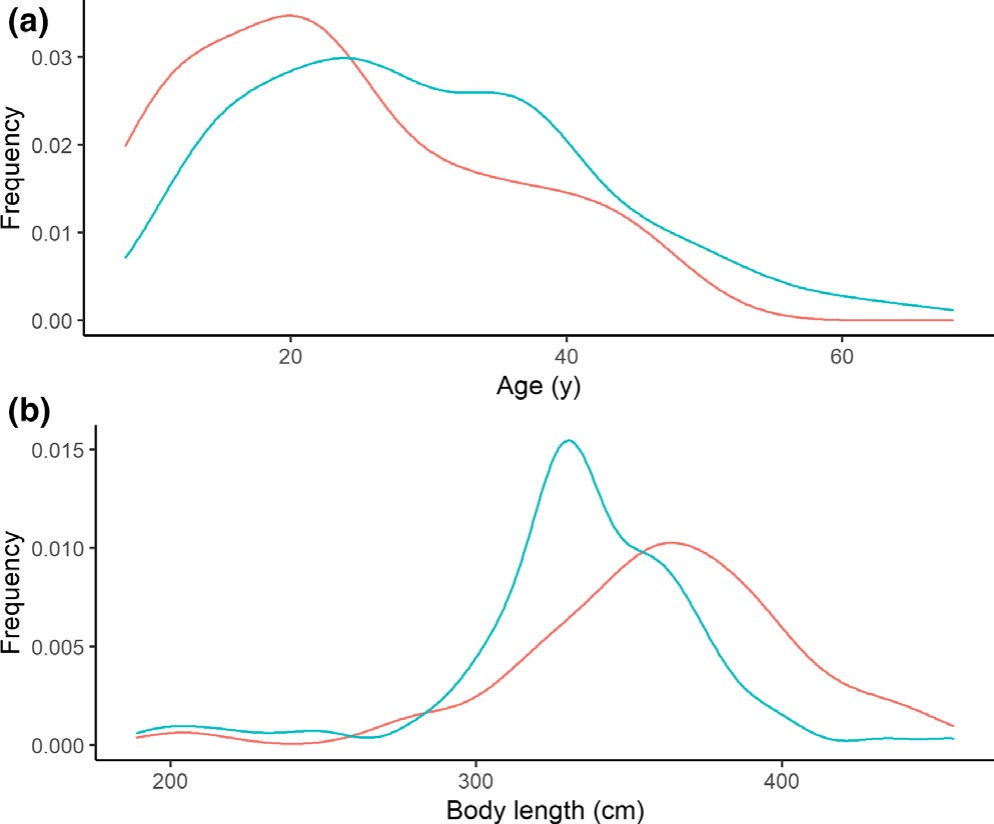
Comparing frequency of female beluga whale ages (top) and lengths (bottom) between BB (red) and HB (blue)

Whales from HB displayed greater ORA (range 1–35, median 8, mean 10.3) than BB (range 1–23, median 6, mean 7.8) (*F*_1,170_ = 3.78, *p* = .05). For age distribution, BB whales (range 8–46, median 21, mean 23.5) were younger than HB whales (range 10–68, median 27, mean 29.5; *F*_1,170_ = 7.77, *p* < .01). For length, BB whales (range 204–447, median 362, mean 359.6) were larger than HB (range 189–455, median 333, mean 333.5; *F*_1,170_ = 12.2, *p* < .001) (Figure 2).

The effect of region (BB and HB) (Step 1) on ORA was assessed in a complete model (ORA ~length + age +region+ (1|year)). Model selection supported two different models (ΔAICc < 1) (Table 1). One of these models found a difference in ORA between regions (ΔAICc = 0.160) and in conjunction with prior knowledge of regional differences, we contrasted BB and HB using separate GLM models to discover any region‐specific age and length relationships (Step 2). For BB beluga whales, length was not a predictor of ORA whereas both age and length were predictors for HB beluga whales (Table 1).

**TABLE 1:** Modeled relationships explaining variation in female beluga whale ORA measured as total corpora counts relative to region (BB (*n* = 20), HB (*n* = 80)), body length (cm), and age (y)

Step 1 complete model					
Model selection table					
Model	df	logLik	AICc	Delta	Weight
ORA ~age + length	4	−565.4	1136.9	0.00	0.402
ORA ~region + age +length	5	−564.4	1137.1	0.16	0.371
ORA ~age	3	−567.4	1138.8	1.89	0.071
ORA ~region + age	4	−567.1	1140.4	3.47	0.071

Comprehensive model				
	Estimate	Std. error	*z* Value	*p*
(Intercept)	0.3844	0.2496	1.540	.1236
Region	0.09650	0.06727	1.434	.1515
Age	0.03562	0.001873	19.016	<.001***
Length	0.002054	0.0006735	3.050	.00229**

Step 2 by regions					
BB model selection table					
Model	df	logLik	AICc	Delta	Weight
ORA ~age	2	−94.351	193.0	0.00	0.73
ORA ~age + length	3	−94.180	195.0	1.99	0.27
ORA ~length	2	−160.474	325.3	132.25	0.00

Comprehensive model				
	Estimate	Std. error	z value	*p*
(Intercept)	0.1902	0.49.40	0.385	.70
Age	0.05776	0.005019	11.508	<2e−16***
Length	0.000792	0.001358	0.583	.56

HB model selection table					
Model	df	logLik	AICc	Delta	Weight
ORA ~age + length	3	−447.937	902.1	0.00	0.950
ORA ~age	2	−451.929	908.0	5.89	0.050
ORA ~length	2	−572.142	1148.4	246.31	0.000

Comprehensive model				
	Estimate	Std. error	z value	*p*
(Intercept)	0.60506	0.29645	2.041	.0412*
Age	0.03549	0.0023337	15.209	<2e−16***
Length	0.001877	0.0008531	2.200	.0278*

Note

Step 1 summarizes model selection and complete model information. Step 2 describes model information for the lowest AIC model from each region (BB and HB) separately. Model selection criteria includes degrees of freedom (df), log‐likelihood (logLik), AICc values, Delta (∆AICc), and AICc weights (weight – relative likelihood of the model).

**FIGURE 3:**
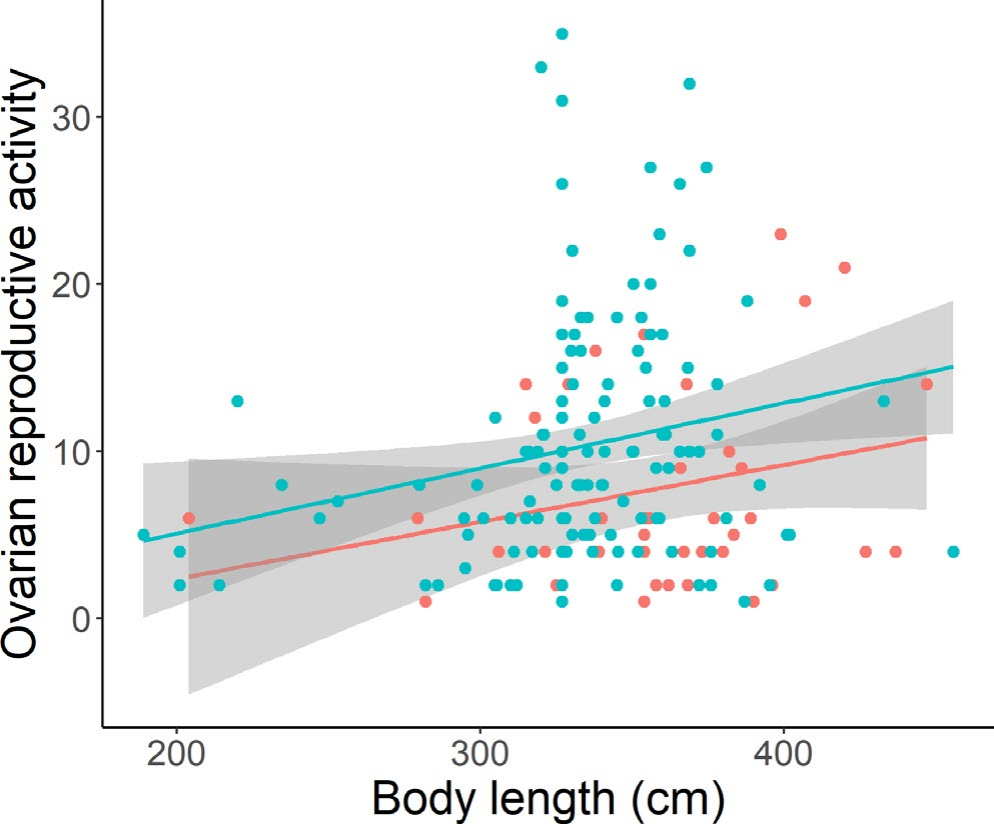
Linear relationship between female ORA and body length for BB (red; ORA = 0.5029 ± 0.0496(length) – 3.749 ± 1.326; *r* ^2^ = .766, *p* < .001) and HB (blue; ORA = 0.3311 ± 0.0367(length) – 0.1331 ± 1.135; *r* ^2^ = .439, *p* < .001) beluga whale populations

Finally, we used partial correlations to account for explained variation only attributable to length. For BB beluga whales, length explained 0.4% of variation in ORA while controlling for age, whereas age explained 63.6% of variation in ORA. For HB beluga whales, length explained 5.7% of the variation in ORA while controlling for age, whereas age explained 41.4% of variation in ORA. For BB beluga whales, the rate of increase in ORA with age was 1.5 times greater than HB (0.50 vs. 0.33 ORA per year, linear regression test for differences in slope: *t* = −2.17, *p* = .031), while the rate of increase in ORA with length did not differ between populations (*t* = 0.53, *p* = .96; Figure 3). However, HB whales had higher ORA for similar body lengths (*t* = 2.95, *p* = .0037). For example, within the 20–40 year age group, BB females had a median length of 368 cm and median ORA of 6 compared to 330 cm and 10 for HB whales. For age (BB: 26.5 y ± 6.2, 21; HB: 29.8 ± 5.9, 75), length (BB: 368.2 ± 51.6; HB: 325.9 ± 44.5), and ORA (BB: 8.48 ± 5.35; HB: 9.96 ± 6.20) effect size was 0.53, 0.88, 0.26, respectively, whereby an effect size (Cohen's *d*) of around 0.5 is considered to be a medium effect size and a *d* of 0.8 or larger is considered to be a large effect size. Length explained 1% of the total variation in ORA for BB beluga whales (0.4%/(0.4% + 63.3%) × 100%) compared to 12% of ORA explained by length for HB (5.7%/5.7% + 41.4%) × 100%).

## 4 DISCUSSION

Population‐level differences in ORA could be an adaptation to environmental selection pressures that vary along latitudinal gradients (Coulon et al., 2008; Orsini et al., 2008; Pauls et al., 2013). Although ORA did not differ among beluga whales along a latitudinal continuum, for the southern population of female beluga whales at the periphery of the species’ geographic range, ORA was more strongly influenced by body size than ORA of populations within the core northern range. Additionally, body size was a greater predictor of ORA for female HB beluga whales living at the southern edge of their distribution compared to BB whales living in the core northern range. If this finding holds for other species facing similar selective pressures from climate warming; then, our results provide critical information on a mechanism of redistribution and underscores limits to opportunities for adaptation in changing environments.

In females, fecundity selection, which selects for traits that increase the number of offspring successfully raised, is a major driver of body size, whereas in males, sexual selection is a major evolutionary force selecting for larger body size (Ralls, 1977). Fecundity selection in females is an adaptation that needs to be balanced with survival (Pincheira‐Donoso & Hunt, 2017). For example, selection for large female body size is counterbalanced by opposing selective forces. First, increased risk from predation, parasitism, or starvation because of their large size (e.g., reduced agility, increased detectability, higher energy requirements, heat stress). Second, a longer developmental time to attain larger size, which may result in a later age of sexual maturity and decreased lifetime reproductive success (Blanckenhorn, 2000).

It is unclear why larger body size among female beluga whales is more strongly correlated with ORA in a population of smaller‐bodied whales living near the southern periphery of their geographic range. It is possible that although larger body size is favored by females in the southern population, due to the high population density, relative to food availability, they may struggle to grow to a size similar to that found in northern areas (Luque & Ferguson, 2010). For the smaller‐bodied whales of southern populations, individual selection may be strong for large females because of the advantages accrued with greater fat storage capability and the associated survival advantages during seasonal food limitation (Lindstedt & Boyce, 1985). Similarly, we would predict that southern populations would select for longer nursing duration due to the advantages provided by greater offspring growth and survival (Beauplet & Guinet, 2007; Matthews & Ferguson, 2015). In contrast, the northern population lives at lower population density and likely without food limitation and thus can grow to a larger body size. Food limitation in southern areas, would contrast with density‐independent limitation through ice entrapments in northern areas, where differences in body size may not provide survival advantages (Heide‐Jørgensen et al., 2002; Luque & Ferguson, 2010).

Another consideration is the contrasting demographic history between the two regions and how long‐term changes in population dynamics can drive differences in ORA. The pristine, pre‐commercial whaling abundance of the BB population was previously estimated to be double that of the most recent population abundance estimate from 1996 of 21,213 beluga whales (Innes et al., 2002; Innes & Stewart, 2002). Although, the population growth trend has been interpreted as increasing, the BB population as a whole is still considered depleted due to past commercial whaling (Hobbs et al., 2020). Similarly, the Cumberland Sound population, also located in the BB region, is considered depleted due to past overharvesting from commercial whaling practices (Sergeant & Brodie, 1975) with a current abundance estimated at 1,381 or 15% of the original estimated population size (Watt et al., 2020). In contrast, the HB population is considered to be possibly the largest in the world, at a minimum size of 54,473 beluga whales (Matthews et al., 2017). Although considerable commercial harvesting of HB beluga whales occurred over the past century (Mitchell & Reeves, 1981), the population is likely at or near carrying capacity (Hammill et al., 2017; Hobbs et al., 2020). Demographic rates differed between the beluga whale populations studied here and research has shown that long‐term population dynamics can not only fluctuate over time, but drive large differences in reproduction (Boyce et al., 2006; Ozgul et al., 2006; this study).

Despite the large number of samples provided by Inuit hunters from across Nunavut, the number of intact and complete female reproductive tracts with ovaries and associated morphometric information was moderate. As a result, we were unable to consider other covariates that may explain ORA variation, such as temporal trends that could be associated with environmental shifts. In addition, since hunters are somewhat selective in the size of harvested whales, there is the possibility of bias in the whales hunted (e.g., health), although we would expect this possible bias to be similar between our two study regions. Another data uncertainty is whether CAs in older females becomes progressively smaller and more difficult to detect (Suydam, 2009). Interpreting ORA of beluga whales is made difficult because of the occurrence of accessory corpora (Burns & Seaman, 1986) and younger females may produce more accessory corpora than older ones (Brodie, 1972; Harrison et al., 1972; Perrin et al., 1984). Greater ORA may also indicate more successful reproduction, resulting in the birth of calves that may or may not survive to reproduce themselves. An unsuccessful pregnancy or calf mortality could result in a shorter reproductive interval and earlier ovulation resulting in a possible bias; similarly, successful pregnancy can result in fewer CAs since ovulation does not occur during the gestation period (Cha et al., 2012). The persistence of CAs provides a measure of the number of successful ovulation events, but it does not provide additional information on reproductive success following birth. We expect these possible biases to be consistent across both regions and are unlikely to affect the comparison of patterns between populations located along a latitudinal continuum; however, it is a limitation of the study. Our statistical assessment of partial correlation assumed linear relationships among ORA, age, and length in order to partition the variance to understand the relationship between ORA and length while controlling for age. However, nonlinearity was evident, particularly for age and ORA, indicating that nonparametric approaches may also be applicable to understand questions unrelated to variance, and should be explored with a larger sample size.

Understanding the evolutionary mechanisms for animal adaptations to shifting environments via changes in life‐history parameters will assist conservation efforts in anticipating and possibly ameliorating future demographic challenges associated with a warming world (Hazen et al., 2013; Sæther et al., 1996; Stockwell et al., 2003). For example, increasing anthropogenic stress from contaminants, noise, and conflicts with fisheries may exacerbate reproductive costs to beluga whales (Mosnier et al., 2015; Norman et al., 2015). Furthermore, contemporary evolution might reduce reproductive success through interactions between population size and strength of selection making most conservation efforts risky unless they can measure and account for changes in fitness (Fernández & Caballero, 2001). More insight is required to understand the complex relationships between changing evolutionary pressures and population dynamics, such as fecundity, individual body growth patterns, sociality, and genetic traits to strengthen conservation efforts, thus ensuring long‐term species persistence.

## CONFLICT OF INTEREST

None declared.

## AUTHOR CONTRIBUTIONS

**Steven H. Ferguson:** Conceptualization (lead); Formal analysis (lead); Funding acquisition (lead); Investigation (lead); Project administration (lead); Supervision (equal); Visualization (lead); Writing‐original draft (lead); Writing‐review & editing (lead). **David J. Yurkowski:** Writing‐review & editing (equal). **Justine Hudson:** Data curation (lead); Investigation (equal); Validation (equal); Writing‐review & editing (equal). **Tera Edkins:** Data curation (equal); Investigation (equal); Validation (equal); Writing‐review & editing (equal). **Cornelia Willing:** Data curation (equal); Investigation (equal); Methodology (lead); Validation (equal); Writing‐review & editing (equal). **Cortney A. Watt:** Project administration (equal); Supervision (equal); Writing‐review & editing (equal).

## Data Availability

All data analyzed in this paper have been uploaded to the Dryad Digital Repository (https://doi.org/10.5061/dryad.hmgqnk9j3).
